# High-throughput sequencing reveals circular RNA hsa_circ_0000592 as a novel player in the carcinogenesis of gastric carcinoma

**DOI:** 10.1042/BSR20181900

**Published:** 2019-06-28

**Authors:** Min Liang, Zhaoyu Liu, Hai Lin, Boyun Shi, Ming Li, Ting Chen, Lingyu Qin, Qiuling Niu, Guifang Yu, Hui Jiang, Xinke Zhou

**Affiliations:** 1 Department of Oncology, the Fifth Affiliated Hospital of Guangzhou Medical University, Guangzhou 510700, China; 2 Department of Oncology, Zhujiang Hospital of Southern Medical University, Guangzhou 510282, China; 3 Department of Central Laboratory, the Fifth Affiliated Hospital of Guangzhou Medical University, Guangzhou 510700, China; 4 Department of Gynaecology, the Fifth Affiliated Hospital of Guangzhou Medical University, Guangzhou 510700, China

**Keywords:** chemical carcinogens, gastric cancer, GES-1-T, hsa_circ_0000592

## Abstract

***Background/Aim:*** Gastric cancer is one of the most common malignant tumors, and its complex pathogenesis has not been fully elucidated. Circular RNAs (circRNAs) are involved in various biological processes and human diseases. However, their exact functional roles and mechanisms of action remain largely unclear. We previously discovered the differential expression of non-coding RNAs (ncRNAs) during the malignant transformation of human gastric epithelial cells. In this study, we investigated the functional roles of a significantly up-regulated circRNA (hsa_circ_0000592) in gastric cancer. ***Methods:*** *N*-methyl-*N*′-nitro-*N*-nitrosoguanidine (MNNG)-induced malignant-transformed gastric epithelial cells (GES-1-T) and normal gastric epithelial cells (GES-1-N) were analyzed by high-throughput circRNA sequencing. The top 15 up-regulated circRNAs in high-throughput sequencing results were further confirmed by qRT-PCR in different gastric epithelial cell lines. The function of the most significant circRNA (hsa_circ_0000592) was investigated by using RNA interference (RNAi) assays, fluorescence *in situ* hybridization analysis (FISH), and bioinformatics prediction methods. ***Results:*** A total of 1509 genes were up-regulated and 3142 genes were down-regulated in GES-1-T cells when compared with GES-1-N cells. When compared with GES-1-N cells, hsa_circ_0000592 was obviously up-regulated in GES-1-T cells, as well as in other gastric cancer cell lines. The silencing of hsa_circ_0000592 mRNA led to a decrease in cell proliferation, cell cycle arrest at the G0/G1 phase, an increased rate of apoptosis, and a reduction in cell migration. Furthermore, FISH showed that hsa_circ_0000592 was mainly located in the cytoplasm, and a bioinformatics analysis suggested that hsa_circ_0000592 might function by sponging multiple miRNAs, and most notably four conserved miRNAs, including miR-139-3p, miR-200, miR-367-3p, and miR-33a-3p. ***Conclusion:*** This study is the first to identify hsa_circ_0000592 as a novel circRNA with a critical role in MNNG-induced gastric cancer. Due to the essential role of hsa_circ_0000592 in gastric carcinoma cells, it may be considered as a potential biomarker for use in diagnosing gastric carcinoma. Our findings provide a new insight into the function of circRNAs in environmental carcinogen-induced gastric cancer.

## Introduction

Gastric cancer is one of the most common malignant tumors. The disease is often diagnosed in its advanced stages due to a lack of distinct symptoms, and thus has a poor prognosis [1]. To date, the complex carcinogenic mechanism of gastric cancer has not been fully elucidated. In recent years, epidemiological data gathered at several locations have identified some commonly encountered chemicals as risk factors for gastric cancer [2,3]; among which, *N*-nitroso compounds (NOCs) have received increased attention [4]. Previous studies have confirmed a strong carcinogenic effect of NOCs in several experimental animals. The genotoxic and carcinogenic effects of NOCs are usually attributable to their ability to damage DNA [5,6]. NOCs and their precursors are abundant in a wide range of foods including pickles [7], processed fish, and meat products [8,9]. Gastrointestinal NOCs are accumulated in the body via either dietary intake or endogenous formation [10], and exposure to NOCs is probably responsible for a significant proportion of gastric cancers. The monofunctional alkylating agent *N*-methyl-*N*′-nitro-*N*-nitrosoguanidine (MNNG), one of the most common NOCs in pickled foods, is known to be a common chemical mutagen and carcinogen that directly damages DNA [11–13].

With the rapid development of molecular medicine, epigenetic mechanisms such as methylation and histone modifications have become research hotspots in molecular genetic studies of gastric cancer [14]. Non-coding RNAs (ncRNAs) are now widely accepted as key regulatory molecules involved in the epigenetic regulation of gene transcription [15]. Several studies have confirmed the role of ncRNAs in the carcinogenesis induced by exogenous chemicals, and shown that the expression levels of multiple miRNAs in cells or tumors are changed after acute or chronic exposure to several known toxic substances [16]. These findings have provided new insights into the study of gastric cancer-related mechanisms. Circular RNAs (circRNAs) exhibit tissue-specific and developmental-specific expression, and play crucial roles in cancer by functioning as miRNA sponges, and thereby affecting the stability of target mRNAs and dynamically regulating mRNA translation [17]. With recent advances in research technologies, and especially the application of high-throughput sequencing technology, a large number of circRNAs have been identified in the eukaryotic transcriptome, some of which are up to 10-fold more abundant than in their linear counterparts [18]. The role of circRNAs in tumorigenesis, and their potential as new clinical diagnostic markers, has gradually become recognized. For example, it has been shown that circRNA ciRS-7 removes or reduces the inhibitory effect of miR-7 on carcinogenic factors including epidermal growth factor receptor (EGFR) [19], insulin receptor substrate 1 (IRS-1), IRS-2 [20], p21-activated protein kinase-1 (Pak1) [21], and Raf1 through competitive binding to miR-7 [22–24], and thus promotes the development of cancer. NOCs are known to be closely associated with the occurrence and development of gastric cancer [25]. Nevertheless, it remains to be investigated whether NOCs directly induce gastric cancer or affect carcinogenesis through stimulation of circRNA expression and the subsequent activation of endogenous signaling pathways. It is therefore logical to speculate that circRNAs might be involved in the pathogenic mechanisms of gastric cancer induced by chemical substances, and especially in light of the important role played by non-coding RNAs in the xenobiotic-related carcinogenic process.

In our preliminary study [26], malignant-transformed human gastric epithelial cells (GES-1), namely GES-1-T cells, were successfully obtained by MNNG induction, and provided a convenient model for use in *in vitro* studies of gastric cancer pathogenesis. It was also demonstrated that small non-coding RNAs (microRNAs) are linked to the MNNG-induced malignant transformation of human gastric epithelial cells (GES-1). In this study, we examined the role of hsa_circ_0000592 in gastric carcinoma cells. Our results provide further insight into the mechanism of chemical carcinogenesis, and suggest potential biomarkers for use in diagnosing gastric carcinoma.

## Methods

### High-throughput sequencing

High-throughput sequencing was performed as follows: ribosomal RNAs (rRNAs) were removed from total RNA, followed by the removal of linear RNAs with RNase R. A standard paired-end library was prepared and reverse transcribed into cDNAs. These cDNAs were end-repaired and then amplified by PCR. A cDNA library of suitable size was selected using quality control methods and loaded onto the sequencing machine. The output sequencing data were obtained in the FASTQ format.

High-throughput sequencing data were analyzed as previously [27,28]. Briefly, the raw data were subjected to filtering, linker sequence removal, and low-quality read processing. The quality of the sequencing was further assessed and the sequencing data for rRNAs were removed to obtain high quality data (clean data). These clean data were then compared with the reference genome by unfusion analysis for unmapped reads. The unmapped reads were further compared with the reference genome by fusion analysis to select circRNA candidates with back-spliced junction reads. CircRNAs were identified after filtering out those candidates without the sequence properties of the splicing site. The selected circRNAs were subjected to further analysis that included annotation, sequence prediction, and the calculation of expression levels.

### Cells and cell culture

Gastric cancer cell lines MKN-28, SGC-7901, MGC-803, HGC-27, and AGS were preserved on a long-term basis in our laboratory. The MNNG-induced malignant-transformed GES-1-T cells and negative control GES-1-N cells were constructed in a preliminary study. MKN-28, SGC-7901, MGC-803, HGC-27, and AGS cells were cultured in RPMI 1640 medium (Invitrogen, Carlsbad, CA, U.S.A.) containing 10% fetal bovine serum (FBS; Gibco-Invitrogen Corp., Carlsbad, CA, U.S.A.). GES-1-T and GES-1-N cells were cultured in minimum essential medium (MEM) (Gibco, Carlsbad, CA, U.S.A.) containing 10% FBS. For re-seeding, cells were grown at 37°C in a humidified incubator with 5% CO_2_. Cells at 70–80% confluence were detached with 0.25% (w/v) trypsin (Gibco, BRL), and passaged at a 1:2 ratio.

### Real-time PCR

Total RNA was extracted using Trizol® reagent (Invitrogen) according the manufacturer’s instructions and contaminating genomic DNA was removed with RNase free DNase I. The concentration and purity of RNA were estimated by measuring absorbance at wavelengths of 260 and 280 nm with a Nanodrop ND-1000 spectrophotometer (Nanodrop Technologies, Montchanin, DE, U.S.A.), and samples with an OD_260/280_ of 1.8–2.0 were used for the next step experiment. RNA integrity was checked by electrophoresis method and observation of two sharp bands for the large and the small subunit rRNAs with the intensity of the larger band being about twice that of the smaller band is indicative of intact RNA. The quantitative real-time reverse transcription-PCR (qRT-PCR) was carried out using the SYBR Premix Ex Taq (TB Green™ Fast qPCR Mix, TakaRa) and the ABI 7500 Real time PCR system as described previously [29,30]. Briefly, qRT-PCR was used to detect gene expression at the transcript level. First, cDNA was reverse transcribed from total RNA by using a Prime-Script® RT kit (TaKaRa, Dalian, China), and the target concentration level was quantified with an Applied Biosystems 7500 Real Time PCR System (Applied Biosystems, Foster City, CA, U.S.A.) and using a SYBR Premix® Ex Taq™ Kit (TaKaRa) and gene-specific primers. The PCR reaction was carried out by mixing 2 μl of cDNA, 1 μl of each upstream and downstream primer, 10 μl of SYBR Premix® Ex Taq™, and 6 μl of ddH_2_O. The reaction conditions were as follows: polymerase activation at 95°C for 30 s, followed by 40 cycles of 95°C for 5 s, and 60°C for 30 s. The relative expression level of the target gene was determined by the 2^−△△Ct^ method and using GAPDH as an internal control. Each sample was measured independently three times. All primers were synthesized by Invitrogen. The primer sequences are listed in Supplementary Table S1.

### RNA interference

Two pairs of silencing interfering RNAs (siRNAs) were designed to knockdown the expression of hsa_circ_0000592; the siRNA were synthesized by Biosense Inc. (Guangzhou, China). The sequences of the siRNAs were as follows: si-hsa_circ_0000592-1 sense 5′-AAUAAGACUUCUUGAAGAC-3′, antisense 5′-GUCUUCAAGAAGUCUUAUU-3′; si-hsa_circ_0000592-2 sense 5′-AGAAGUCUUAUUGCCAGCA-3′; antisense 5′-UGCUGGCAAUAAGACUUCU-3′; negative control siRNA sense 5′-UUCUCCGAACGUGUCACGU-3′; and antisense 5′-ACGUGACACGUUCGGAGAA-3′. GES-1-T and MKN-28 cells were seeded into six-well plates and incubated until the cells reached 30% confluence. Cell transfection was preformed using Lipofectamine™ 2000 (Invitrogen) according to the manufacturer’s instructions. After transfection, the culture medium was replaced with fresh DMEM containing 10% FBS, and the cells were incubated for 6 h. Total RNA was extracted after 48 h to measure the interference efficiency of siRNA by qRT-PCR.

### Cell proliferation assay

After transfection, the *in vitro* proliferation of cells in each group was assayed by using a Cell Counting Kit-8 (CCK-8; Dojindo, Tokyo, Japan). Cells (3 × 10^3^ in 100 ml of cell medium) were transferred into the wells of 96-well plates and cultured for 24 h under normal conditions; after which, they were transfected with siRNAs. After incubation for 24, 48, 72, or 96 h, 10 μl of CCK-8 solution was added to each well. Next, the plates were returned to the incubator for 4 h, and the absorbance (*A*) of each well at 450 nm was measured with a Synergy 2 microplate reader (BioTek, Winooski, VT, U.S.A.). Cell viability (percent of control) was calculated using the following formula: (OD_test_ − OD_blank_) / (OD_control_ − OD_blank_), where OD_test_, OD_control_, and OD_blank_ denote the absorbance value of the experimental group, negative control group, and the well containing only medium.

### Cell cycle analysis

Cells were seeded into six-well plates and incubated for 24 h prior to transfection. After 48 h of transfection, the cells were harvested by trypsinization and washed twice with phosphate-buffered saline (PBS); after which they were mixed with 1 ml of 70% (v/v) iced ethanol, fixed overnight at 4°C, washed twice with 2 ml of pre-chilled 0.1% Triton-X-100, and resuspended in PBS. An appropriate amount of RNase A was added to a final concentration of 200 μg/ml, and the mixture was incubated in a water bath at 37°C for 30 min. Propidium iodide (PI) was then added to a final concentration of 20 μg/ml, and the mixture was incubated in a 37°C water bath in the dark for 30 min. The cell cycle was analyzed by flow cytometry (FCM) (FACScan; Becton Dickinson, Franklin Lakes, NJ, U.S.A.), and the resultant data were analyzed using FlowJo software (Tree Star Inc., Ashland, OR, U.S.A.).

### Flow cytometry apoptosis assay

Cell apoptosis was detected using an Annexin V-FITC/PI kit (KeyGen Biotech, Nanjing, China). Briefly, cells were collected by trypsin digestion at 48 h after transfection. The cells were then washed twice with ice-cold PBS, and successively mixed with 500 μl of binding buffer, 5 μl of Annexin V-FITC, and 5 μl of propidium iodide (PI). The mixture was allowed to react in the dark at room temperature for 15 min. Cell apoptosis was measured by FCM (FACScan) within 1 h, and the proportion of apoptotic cells was recorded. Each sample was assayed three times.

### CCK-8 cell adhesion to extracellular matrix

The 96-well plates used for testing were pre-coated overnight with 10 μg/ml fibronectin (FN, 70 μl/well) at 4°C, washed with PBS, and then blocked with 1% bovine serum albumin (BSA) at 37°C for 1 h. Cells in their exponential phase were digested, resuspended in serum-free medium, counted with a hemocytometer, and suspended at a concentration of 5 × 10^5^/ml. Aliquots of suspended cells were inoculated into the FN-pre-coated 96-well plates (5000 cells per well). Three wells were prepared for each group. The plates were incubated at 37°C for 1 h, and then washed with PBS to remove non-adherent cells. The numbers of adherent cells were determined with the CCK-8 assay.

### Scratch wounding assay

GES-1-T and MKN-28 cells in all groups were seeded into six-well plates at a density of 5 × 10^5^ cells/well, and the incubated at 37°C until they reached 100% confluence. The centre of the cell monolayer was scraped with a sterile pipette tip and washed three times with PBS to create a straight, cell-free zone (gap) of constant width. Cells were then transferred into serum-free medium. Wound closure was monitored and photographed at 0 and 24 h with an inverted microscope (Olympus, Tokyo, Japan). Images were analyzed using ImageJ software (http://rsb.info.nih.gov/ij/), and the relative area ratios of cell migration were calculated using the following formula: relative area ratio of cell migration = (scratch wound area after 24 h of culture/initial scratch wound area) × 100%.

### Fluorescence *in situ* hybridization analysis

Reagents were purchased from Biosense (Guangzhou, China). The oligonucleotide probe for hsa_circ_0000592 (5′-TGCTGGCAATAAGACTTCTTGAAGA-3′) was synthesized by Biosense. Cells in each group were fixed in 4% paraformaldehyde for 20 min, washed with distilled water, permeabilized twice with 0.5% TritonX-100 for 20 min, washed twice with 2 × SSC, dehydrated through a series of pre-chilled ethanol solutions (70, 85, and 100%), and finally air-dried at room temperature. The probe and buffer were mixed at a ratio of 2:8 in the dark. Cells were incubated with 10 μl of probe mixture overnight at 37°C. Next, the cells were washed three times with preheated (43°C) 50% formamide/2 × SSC for 5 min, 2 × SSC for 5 min each, counter-stained with DAPI (4′,6-diamidino-2-phenylindole) containing the quencher Vectorshield (Vector Laboratories, Burlingame, CA, U.S.A.), and observed under a fluorescence microscope (Olympus) after 20 min. Images were captured with a photometrics SenSys CCD1400E camera and Metamorph 4.6.3 (Universal Imaging Crop), and analyzed using Photoshop CS 9.0. Positive and negative controls were analyzed by Fluorescence *in situ* hybridization analysis (FISH) as described above. Blank controls were analyzed by FISH performed without the probe.

### Analysis of the cytoplasmic and nuclear distribution of hsa_circ_0000592

Nuclear and cytoplasmic RNAs were extracted using an Ambion Paris kit (Ambion, Austin, TX, U.S.A.) according to the manufacturer’s instructions. Briefly, the cells were harvested by trypsinization, washed with PBS, resuspended in cold fractionation buffer, incubated on ice, and then centrifuged. The cytoplasmic fraction was collected, while the nuclear pellet was lysed with disruption buffer; after which, 2 × lysis/binding solution was added to each fraction. RNA was isolated from separate lysates by the addition of ethanol, and filtered through a cartridge. The nuclear and cytoplasmic RNAs (800 ng) were then converted to cDNA and analyzed by qRT-PCR.

### Statistical analysis

All results were analyzed using SPSS for Windows, Version 16.0 (SPSS Inc., Chicago, IL, U.S.A.) and the Prism statistical software package (Version 5.0, Graphpad Software Inc.). Unpaired *t*-tests (for data with a normal distribution) or Mann–Whitney *U* tests (for data with a non-normal distribution) were used to compare two groups, and multiple group comparisons were analyzed with one-way ANOVA. All statistical results are presented as the mean ± standard deviation of data obtained from at least three independent experiments. A two-sided *P*-value < 0.05 was regarded as statistically significant.

## Results

### High-throughput circRNA sequencing

**Figure 1:**
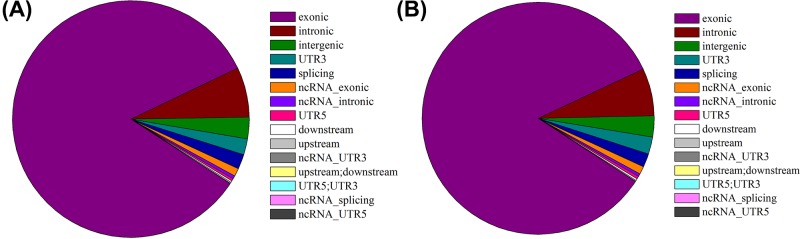
High-throughput circRNA sequencing analysis of the distribution of circRNAs in GES-1-T and GES-1-N cells The distribution of circRNA genes in the GES-1-T genome (**A**). The distribution of circRNA genes in the GES-1-N genome (**B**).

In our preliminary study [26], malignant-transformed GES-1-T cells were successfully generated by MNNG induction. In this study, GES-1-T and normal control (GES-1-N) cells were analyzed by high-throughput circRNA sequencing. After processing raw data from which low quality, linker, and other contaminating sequence reads had been removed, totals of 194015450 and 187645342 filtered reads were obtained for GES-1-T and GES-1-N cells, respectively. Totals of 193704604 (99.84%) and 187445976 (99.89%) effective reads were obtained after the removal of rRNA sequences, and then compared with the reference genome to predict circRNA candidates with back-spliced junction reads. CircRNAs were identified after filtering out those candidates without the sequence properties of a reverse splicing site. The identified circRNA sequences were queried across several databases with ANNOVAR software to annotate their functional elements, including exons, introns, splicing sites, 5′UTR, 3′UTR, and gene gaps. The locations of circRNAs in the genome (Figure 1A,B) were determined based on the location of the junction sequences adjacent to the splicing sites of circRNAs. Our results revealed large numbers of circRNAs in both GES-1-T and GES-1-N cells. The full length of circRNAs was predicted based on the splicing sites as previously described [24]. The number of circRNA reads was obtained, and the reads per million mapped reads (RMP) was calculated as the expression value of circRNAs [32]. The circRNAs with low expression values (SRPBM < 50) in all samples were removed, and circRNAs with a | log2 (fold change) > 1 and *q*-value < 0.05 were considered as genes with significantly differential expression. Totals of 1509 up-regulated and 3142 down-regulated genes in GES-1-T cells were identified by comparison with GES-1-N cells.

### Elevated levels of circRNA hsa_circ_0000592 in gastric cancer cells

**Figure 2:**
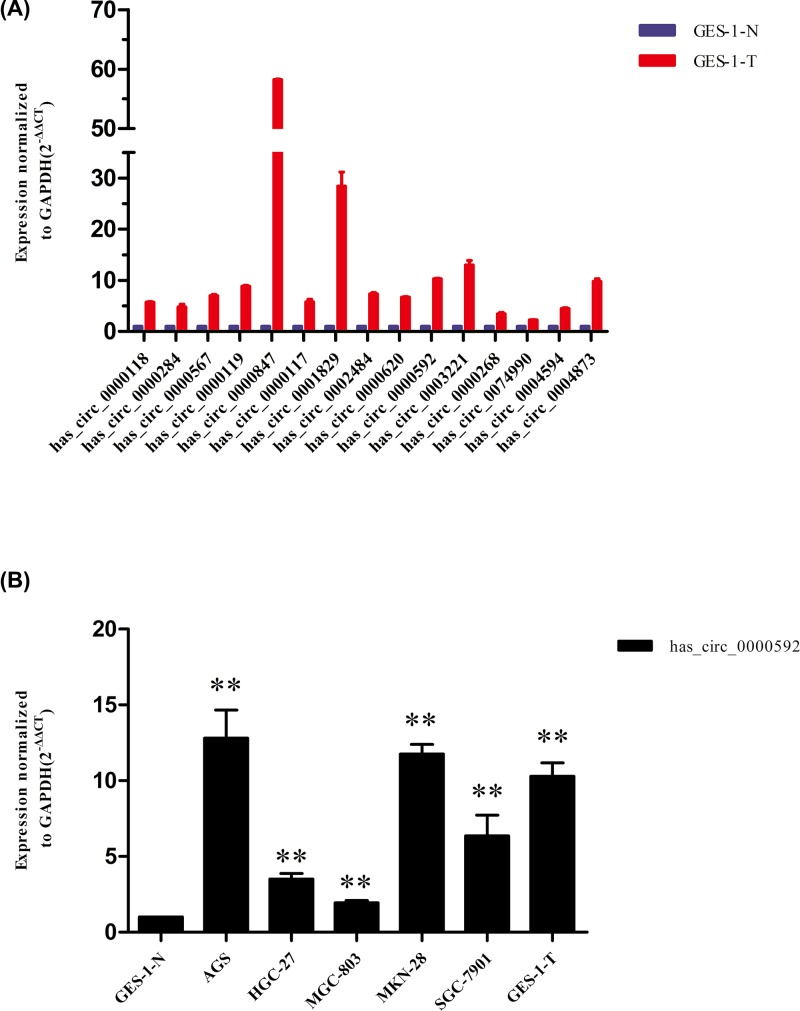
Up-regulating of hsa_circ_0000592 expression in MNNG-induced malignant-transformed GES-1-T cells and several gastric cancer cell lines The relative expression of fifteen circRNAs were validated using qRT-PCR, and hsa_circ_0000592 was significantly up-regulated in GES-1-T cells (**A**). The relative expression level of hsa_circ_0000592 in normal gastric mucosal epithelial GES-1-N cells and several gastric cancer cell lines (**B**) circRNAs were normalized to GAPDH expression, data represent the means of three independent experiments, and the relative expression was calculated using the 2^−ΔΔCt method^. ** *P* < 0.01.

A total of 15 circRNAs with the highest degrees of up-regulation in GES-1-T cells were further analyzed by qRT-PCR in order to identify circRNAs that might be essential for the pathogenesis of chemical carcinogen-induced gastric cancer. Seven circRNAs with the most pronounced up-regulation in GES-1-T cells (has_circ_0000119, has_circ_0000847, has_circ_0000829, has_circ_0002484, hsa_circ_0000592, has_circ_0003221, and has_circ_0004873) were identified by comparison with control GES-1-N cells (Figure 2A). CircRNA hsa_circ_0000592, which is highly conserved in mammals, was selected for further analysis. The level of hsa_circ_0000592 in GES-1-T cells was 10.299-fold higher than that in control GES-1-N cells (*P* < 0.01), and was also higher in gastric cancer AGS, SGC-7901, MGC-803, MKN-28, and HGC-27 cells. The level of hsa_circ_0000592 in MKN-28 cells was 11.763-fold higher than that in control GES-1-N cells (*P* < 0.01, Figure 2B). These results showed that hsa_circ_0000592 levels were increased in both malignant-transformed GES-1-T cells and other gastric cancer cell lines, suggesting that has_circ_0000592 might play an important role in the development of gastric cancer.

### *In vitro* functional study of gastric cancer cells with abnormal levels of circRNAhsa_circ_0000592

**Figure 3:**
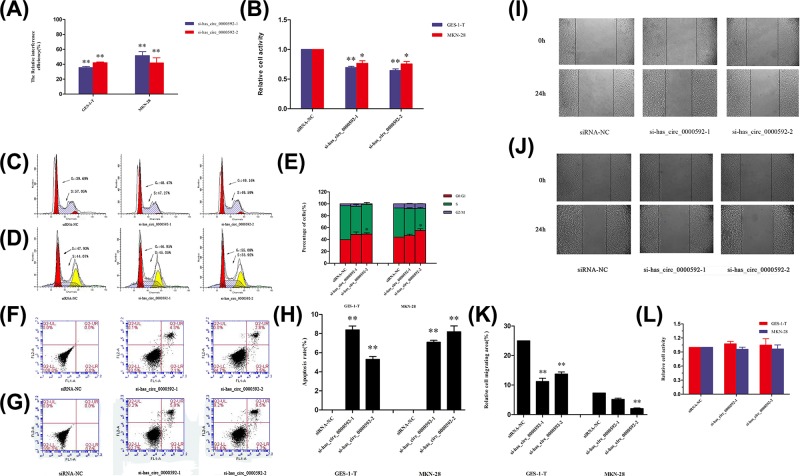
Up-regulation of hsa_circ_0000592 promoted the proliferation and migration of GES-1-T and MKN-28 cells The transfection efficiencies of si-hsa_circ_0000592-1 and si-hsa_circ_0000592-2 in GES-1-T and MKN-28 cells were significantly different from that in the siRNA-NC group (**A**). CCK-8 assays of cell proliferation at 48 h after si-hsa_circ_0000592-1 or si-hsa_circ_0000592-2 transfection. Error bars represent the SD, and ** signifies a *P*-value < 0.01 when compared with the siRNA-NC group (**B**). Flow cytometric analysis of the cell cycle of GES-1-T cells with reduced hsa_circ_0000592 levels (**C**). Flow cytometric analysis of the cell cycle of MKN-28 cells with reduced hsa_circ_0000592 levels (**D**). The G_0_/G_1_, S, and G_2_/M phase distributions of GES-1-T and MKN-28 cells with reduced hsa_circ_0000592 levels. Error bars represent the SD, and * signifies a *P*-value < 0.05 when compared with the siRNA-NC group (**E**). PI staining showing how si-hsa_circ_0000592-1 and si-hsa_circ_0000592-2 function in the cell cycle of GES-1-T cells (**F**). PI staining showing how si-hsa_circ_0000592-1 and si-hsa_circ_0000592-2 function in the cell cycle of MKN28 cells (**G**) the role of si-hsa_circ_0000592 in regulating the GES-1-T and MKN-28 cell cycles. Error bars represent the SD, and * signifies a *P*-value < 0.05 when compared with the siRNA-NC group (**H**). Representative images of the scratch wounding assay used to detect the migration of GES-1-T cells transfected with siRNA at 0 and 24 h (**I**). Representative images of the scratch wounding assay used to detect the migration of MKN-28 cells transfected with siRNA at 0 and 24 h (**J**). The migration of GES-1-T and MKN28 cells at 48 h after transfection with si-hsa_circ_0000592-1 or si-hsa_circ_0000592-2. Error bars represent the SD, and * signifies a *P*-value < 0.05 when compared with the siRNA-NC group (**K**). CCK-8 assay detecting the adhesion ability of cells at 48 h after transfection with si-hsa_circ_0000592-1 or si-hsa_circ_0000592-2 (**L**).

To investigate the effects of circRNA hsa_circ_0000592 on the function of gastric cancer cells, two siRNAs (Supplementary Table S2) targeting hsa_circ_0000592 were transfected into GES-1-T and MKN-28 cells, and the levels of hsa_circ_0000592 expression in both cell lines were measured by qRT-PCR after 48 h. Transfection of si-hsa_circ_0000592-1 or si-hsa_circ_0000592-2 into GES-1-T cells inhibited hsa_circ_0000592 expression by 35.5 ± 1.6% and 42.1 ± 1.0%, respectively, when compared with transfection with the siRNA-NC (*P* < 0.01). Transfection of si-hsa_circ_0000592-1 or si-hsa_circ_0000592-2 into MKN-28 cells inhibited has_circ_0000592 expression by 51.5 ± 5.4% and 41.8 ± 6.9%, respectively (*P* < 0.01, Figure 3A). Therefore, si-hsa_circ_0000592-1 and si-hsa_circ_0000592-2 were selected for further study.

In order to study the effect of reduced hsa_circ_0000592 levels on cell proliferation, GES-1-T and MKN-28 cells were transfected with si-hsa_circ_0000592-1 or si-hsa_circ_0000592-2, and cell viability was assayed using a CCK-8 assay kit after 48 h. The results showed that the viability of GES-1-T cells (32.2 ± 1.0% and 29.9 ± 1.3%, respectively) and MKN-28 cells (31.5 ± 1.8% and 31.1 ± 1.7%) in the si-hsa_circ_0000592-2 and si-hsa_circ_0000592-1 transfection groups was significantly lower than that in the siRNA-NC transfection group (*P* < 0.05, Figure 3B), suggesting that hsa_circ_0000592 expression affected the proliferation of both GES-1-T and MKN-28 cells.

The cell cycles of GES-1-T and MKN-28 cells with reduced hsa_circ_0000592 expression were analyzed by FCM to investigate whether the effect of hsa_circ_0000592 on cell proliferation was mediated through its effect on the cell cycle. Our results showed that the proportion of GES-1-T cells in G_0_/G_1_ phase in the si-hsa_circ_0000592-2 group (49.16%) was significantly higher than in the siRNA-NC group (39.69%, *P* < 0.05, Figure 3C,E). Moreover, the proportion of MKN-28 cells in G_0_/G_1_ phase increased from 47.93% to 55.08% (*P* < 0.05, Figure 3D,E), suggesting that hsa_circ_0000592 helps to facilitate cell cycle progression.

The apoptosis rates of GES-1-T and MKN-28 cells transfected with siRNA were also analyzed by FCM. The apoptosis rates of GES-1-T cells in the si-hsa_circ_0000592-2 and si-hsa_circ_0000592-1 group (6.4% and 5.3%, respectively) were significantly increased when compared with that in the siRNA-NC group (*P* < 0.05, Figure 3F,H). The apoptosis rate of MKN-28 cells in both siRNA groups was also increased after transfection, suggesting that hsa_circ_0000592 affects cell viability.

The migratory ability of GES-1-T cells and MKN-28 cells transfected with siRNAs was further assessed by the scratch wounding assay. After 24 h, the relative migration areas of GES-1-T cells in the si-hsa_circ_0000592-1 and si-hsa_circ_0000592-2 groups were reduced by 11.22% and 13.73%, respectively (Figure 3I,K) when compared with that in the siRNA-NC group (*P* < 0.05). The relative migration areas of MKN-28 cells in the si-hsa_circ_0000592-1 and si-hsa_circ_0000592-2 groups were also significantly reduced (11.22% and 13.73%, respectively, Figure 3J,K).

The adherence ability of cells transfected with siRNAs was determined by the CCK-8 assay. There was no significant difference in the adherence ability of GES-1-T and MKN-28 cells in the siCircRNA_RNA-1 group, siCircRNA_RNA-2, and siRNA-NC group (Figure 3L), suggesting that the regulatory effect of hsa_circ_0000592 on cell migration was not mediated by its effect on cell adhesion.

### Predominant cytoplasmic localization of hsa_circ_0000592

**Figure 4:**
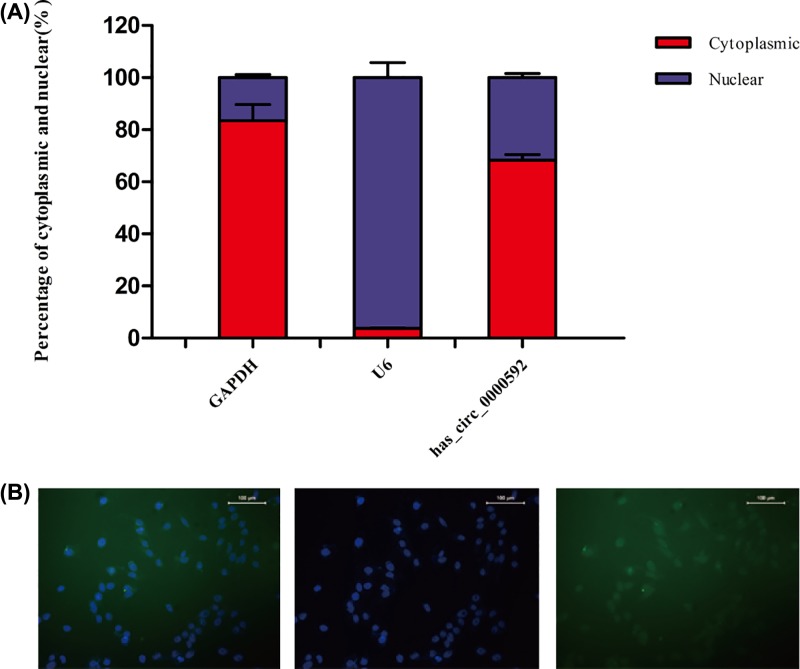
Cytoplasmic and nuclear distribution of hsa_circ_0000592 in GES-1-T cells as detected by qRT-PCR performed with two pairs of specific primers Error bars represent the SD (**A**). FISH analysis of hsa_circ_0000592 levels in GES-1-T cells. Green fluorescence indicates hsa_circ_0000592 (**B**).

In eukaryotic cells, RNA modification and regulation of transcription both occur in the nucleus, whereas protein translation, as well as protein and RNA metabolism occur in the cytoplasm. Therefore, an analysis of the cytoplasmic/nuclear distribution of hsa_circ_0000592 is essential for understanding the function of hsa_circ_0000592. We quantified the relative hsa_circ_0000592 levels in the nucleus and cytoplasm of GES-1-T cells by qRT-PCR, and used U6 and GAPDH as internal controls. The cytoplasmic and nuclear CT values were obtained, and the cytoplasmic/nuclear RNA ratio was calculated as 2 ∧ (cytoplasmic CT – nuclear CT). As shown in Figure 4A, the cytoplasmic/nuclear RNA ratio of U6 was close to 0, indicating its location in the nucleus, whereas the cytoplasmic/nuclear RNA ratio of GAPDH was ∼5, which is consistent with its primary location in the cytoplasm (82%). Our results showed that the hsa_circ_0000592 level in the cytoplasm was at least two-fold higher than that in the nucleus.

The levels of hsa_circ_0000592 in GES-1-T cells were further measured by the FISH assay. Consistent with the qRT-PCR results, the FISH assay show that hsa_circ_0000592 was primarily located in the cytoplasm (green area, Figure 4B), suggesting that hsa_circ_0000592 mainly exerts its function in the cytoplasm.

### A preliminary study of the mechanism of hsa_circ_0000592 activity in gastric cells

**Figure 5:**
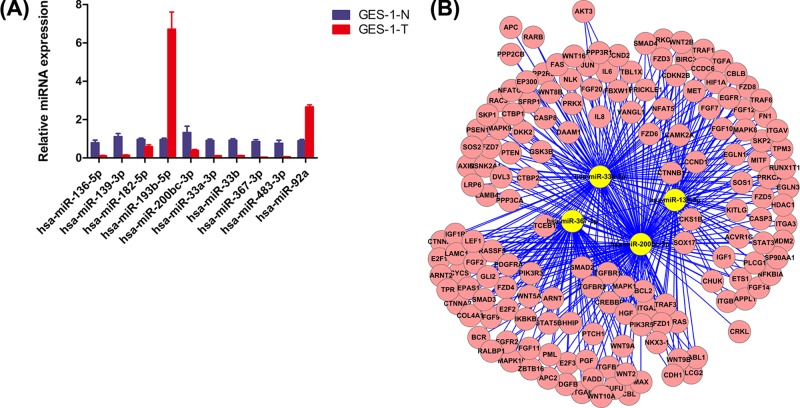
MiRNAs that were negatively correlated with hsa_circ_0000592 levels qRT-PCR analyses of the levels of 12 miRNAs in GES-1-T and GES-1-N cells. Error bars represent the SD, and ** signifies a *P*-value < 0.01 when compared with GES-1-N cells (**A**). The distribution of cancer-related mRNAs that are targets of miR-139-3P, miR-200bc-3p, miR-33a-3P, and miR-367-3P (**B**).

We found that the proliferative ability of cells with a reduced hsa_circ_0000592 level was decreased, which coincided with a mild accumulation of cells in G_0_/G_1_ phase. Because both hsa_circ_0000592 and miRNA are localized in the cytoplasm, we hypothesized that hsa_circ_0000592 might function through competitive binding to downstream miRNAs accomplished by competing with endogenous RNA (ceRNA). A bioinformatics prediction made using software such as TargetsScan, miRanda, and Picta identified 134 miRNAs that might interact with hsa_circ_0000592. Next 12 miRNAs (including miR-139-3p, miR-200b, and miR-200c) were subjected to both a qRT-PCR and bioinformatics analysis. Our results showed that the levels of four miRNAs (including miRNA-139) in GES-1-T cells were markedly reduced when compared with their levels in the negative control group (Figure 5A). Futhermore, the levels of those miRNAs were negatively correlated with the level of hsa_circ_0000592, and were associated with several tumor-related miRNAs (Figure 5B), suggesting the possible involvement of ceRNA with those miRNAs and hsa_circ_0000592. However, these findings require further verification.

## Discussion

CircRNA is currently a hot research topic. CirRNAs were first identified in 1993 and thought to play essential roles in numerous biological processes. CircRNAs have been shown to be highly correlated with multiple tumors, and play an indispensable role in tumor development and progression. However, little is known about the roles played by circRNA in environmental carcinogen-induced malignant transformation, even though exposure to environmental carcinogens is one of the most important causes of gastric cancer. Therefore, determining the roles of circRNAs and the mechanisms involved in environmental carcinogen-induced gastric cancer may provide new ideas for diagnosing and treating that malignancy.

In this study, high-throughput RNA sequencing and the qRT-PCR showed a significant up-regulation of hsa_circ_0000592 expression in GES-1-T cells when compared with hsa_circ_0000592 expression in GES-1-N cells. Furthermore, redundant expression of hsa_circ_0000592 was also detected in other gastric cancer cell lines, including AGS, SGC-7901, MKN-28, and HGC-27. These findings suggest that hsa_circ_0000592 may serve as a cancer facilitator during MNNG-induced gastric cell transformation. Knockdown, subcellular localization, and interaction assays were performed to evaluate the effects of hsa_circ_0000592 on the proliferation, cell cycle, and apoptosis of gastric cancer cells. Our results showed that knocking down the expression level of hsa_circ_0000592 suppressed the proliferation of gastric cancer cells by forcing Go/G1 phase cell cycle arrest, and increasing the apoptosis rate. Further experiments revealed that hsa_circ_0000592 is mainly located in the cytoplasm, and might function by interacting with several miRNAs.

In recent years, circRNAs have received increased attention from RNA researchers due to their ubiquitous existence in mammalian cells. Previous studies have shown that the distribution of circRNA in cells may be associated with different functions. CircRNAs in the nucleus might be involved in chromatin remodeling, while circRNAs in the cytoplasm might interact with miRNA and subsequently affect the expression of downstream mRNA molecules. To determine the specific function of hsa_circ_0000592 in gastric cancer cells, we first determined its intracellular localization in those cells. Our results showed that hsa_circ_0000592 was predominantly located in the cytoplasm, which suggested that hsa_circ_0000592 probably functions in the cytoplasm. Consistent with our results, previous studies have also found that circRNAs are mainly distributed in the cytoplasm of eukaryotic cells due to special variable shearing [17,31], and only a few circRNAs are located in the nucleus [32]. Several circRNAs that function in the cytoplasm have been identified. For example, has_circ_0001946, a novel circRNA found in lung adenocarcinoma, is mainly present in the cytoplasm, and acts as a ceRNA by targeting miRNA-135a; moreover, it also plays a regulatory role in Wnt/β-catenin signaling. Xiaoxu et al. found that circAKT3, a circRNA originating from exons 8, 9, 10, and 11 of the AKT3 gene, is mainly distributed in the cytoplasm, and can up-regulate PIK3R1 expression to enhance cisplatin resistance in gastric cancer cells by suppressing miR-198 expression. CircLARP4, a cytoplasmic cirRNA, was found to inhibit the proliferation and invasion of gastric cancer cells by sponging miR-424-5p and regulating LATS1 expression.

CircRNAs can have special biological functions due to their unique structure [18]. Studies have shown that circRNAs can regulate the levels of host gene expression [32], mediate translation by interacting with RNA binding proteins, exert *cis*-transcriptional regulation [31], and regulate alternative splicing [33]. It is well known that circRNAs can function as both ceRNA and novel miRNA activity regulatory molecules in post-transcriptional gene regulation [34]. Cerebellar degeneration-related protein 1 (CDR1as) is known to be associated with miR-7 [35], which can regulate the occurrence of several tumors including lung cancer [36], gastric cancer [37], and malignant Schwannoma by inhibiting the expression of its target genes, such as *EGFR* [38,39], insulin-like growth factor receptor (*IGF1R*) [37], and activated Cdc42-associated tyrosine kinase 1 (*ACK1*) [38]. In this study, we found that several miRNAs, including miRNA139-3P, miR-200bc-3p, miR-33a-3P, and miR-367-3P, were negatively correlated with the level of hsa_circ_0000592 expression, and specifically targeted a variety of cancer-related mRNAs. Hansen et al. [40] showed that CDR1as is degraded by miR-671 because it is fully complementary to miR-671 instead of miR-7-mediated RISC, suggesting that miR-671 can indirectly regulate miR-7 activity by reducing the level of CDR1as. When compared with other types of ceRNAs, circRNAs are expressed at high levels and exhibit strong stability in cells with high levels ceRNA activity, and thus more miRNA binding sites. Under those conditions, circRNAs can more effectively regulate miRNA levels by rapidly and stably binding or releasing large amounts of miRNA within cells [16]. However, it needs to be further investigated whether circRNAs can act as miRNA sponges and regulate the occurrence and development of cancers by competitively mediating the transcription of miRNAs such as miR-139-3P, and thereby the expression of their downstream target genes.

In summary, this screening and functional study identified hsa_circ_0000592 as a novel biomarker for gastric cancer, and suggested that hsa_circ_0000592 might serve as new target for preventing and treating MNNG-induced gastric cancer.

## Supporting information

**Supplemental Table S1:** RNA Primer sequences

**Supplemental Table S2:** siRNA sequences

**Supplemental Table S3:** 

## Abbreviations

CCK-8: Cell Counting Kit-8
CDR1as: cerebellar degeneration-related protein 1
ceRNA: competitive endogenous RNA
circRNA: circular RNA
EGFR: epidermal growth factor receptor
FBS: fetal bovine serum
FCM: flow cytometry
FISH: fluorescence in situ hybridization analysis
FN: fibronectin
IRS-1: insulin receptor substrate 1
MNNG: *N*-methyl-*N*′-nitro-*N*-nitrosoguanidine
ncRNA: non-coding RNA
NOC: *N*-nitroso compound
Pak1: p21-activated protein kinase-1
PBS: phosphate-buffered saline
PI: propidium iodide
qRT-PCR: quantitative real-time reverse transcription-PCR
RNAi: RNA interference
rRNA: ribosomal RNA
siRNA: silencing interfering RNA

## References

[1] Torre L.A., Bray F., Siegel R.L., Ferlay J., Lortet-Tieulent J. and Jemal A. (2015) Global cancer statistics, 2012. CA Cancer J. Clin. 65, 87–108 10.3322/caac.21262 25651787

[2] Forman D. and Burley V.J. (2006) Gastric cancer: global pattern of the disease and an overview of environmental risk factors. Best Pract. Res. Clin. Gastroenterol. 20, 633–649 16997150 10.1016/j.bpg.2006.04.008

[3] Garcia-Perez J., Lopez-Cima M.F., Perez-Gomez B., Aragones N., Pollan M., Vidal E. (2010) Mortality due to tumours of the digestive system in towns lying in the vicinity of metal production and processing installations. Sci. Total Environ. 408, 3102–3112 10.1016/j.scitotenv.2010.03.051 20427078

[4] Keszei A.P., Goldbohm R.A., Schouten L.J., Jakszyn P. and van den Brandt P.A. (2013) Dietary N-nitroso compounds, endogenous nitrosation, and the risk of esophageal and gastric cancer subtypes in the Netherlands Cohort Study. Am. J. Clin. Nutr. 97, 135–146 10.3945/ajcn.112.043885 23193003

[5] Hebels D.G., Sveje K.M., de Kok M.C., van Herwijnen M.H., Kuhnle G.G., Engels L.G. (2011) N-nitroso compound exposure-associated transcriptomic profiles are indicative of an increased risk for colorectal cancer. Cancer Lett. 309, 1–10 10.1016/j.canlet.2011.05.007 21669488

[6] Sedgwick B., Bates P.A., Paik J., Jacobs S.C. and Lindahl T. (2007) Repair of alkylated DNA: recent advances. DNA Repair (Amst.) 6, 429–442 10.1016/j.dnarep.2006.10.005 17112791

[7] Luo R.H., Zhao Z.X., Zhou X.Y., Gao Z.L. and Yao J.L. (2005) Risk factors for primary liver carcinoma in Chinese population. World J. Gastroenterol. 11, 4431–4434 10.3748/wjg.v11.i28.4431 16038048 PMC4434676

[8] De Jonge P.J., Wolters L.M., Steyerberg E.W., Van Dekken H., Kusters J.G., Kuipers E.J. (2007) Environmental risk factors in the development of adenocarcinoma of the oesophagus or gastric cardia: a cross-sectional study in a Dutch cohort. Aliment. Pharmacol. Ther. 26, 31–39 17555419 10.1111/j.1365-2036.2007.03344.x

[9] Takezaki T., Gao C.M., Wu J.Z., Ding J.H., Liu Y.T., Zhang Y. (2001) Dietary protective and risk factors for esophageal and stomach cancers in a low-epidemic area for stomach cancer in Jiangsu Province, China: comparison with those in a high-epidemic area. Jpn. J. Cancer Res. 92, 1157–1165 10.1111/j.1349-7006.2001.tb02135.x 11714439 PMC5926655

[10] Risch H.A. (2003) Etiology of pancreatic cancer, with a hypothesis concerning the role of N-nitroso compounds and excess gastric acidity. J. Natl. Cancer Inst. 95, 948–960 10.1093/jnci/95.13.948 12837831

[11] Abe M., Yamashita S., Kuramoto T., Hirayama Y., Tsukamoto T., Ohta T. (2003) Global expression analysis of N-methyl-N’-nitro-N-nitrosoguanidine-induced rat stomach carcinomas using oligonucleotide microarrays. Carcinogenesis 24, 861–867 10.1093/carcin/bgg030 12771029

[12] Raphael K.R., Sabu M., Kumar K.H. and Kuttan R. (2006) Inhibition of N-Methyl N’-nitro-N-nitrosoguanidine (MNNG) induced gastric carcinogenesis by Phyllanthus amarus extract. Asian Pac. J. Cancer Prev. 7, 299–302 16839226

[13] Grossi M.R., Berni A., Pepe G., Filippi S., Mosesso P., Shivnani A.A. (2012) A comparative study of the anticlastogenic effects of chlorophyllin on N-methyl-N’-nitro-N-nitrosoguanidine (MNNG) or 7,12-dimethylbenz (alpha) anthracene (DMBA) induced micronuclei in mammalian cells in vitro and in vivo. Toxicol. Lett. 214, 235–242 10.1016/j.toxlet.2012.08.023 22985524

[14] Kung J.T., Colognori D. and Lee J.T. (2013) Long noncoding RNAs: past, present, and future. Genetics 193, 651–669 10.1534/genetics.112.146704 23463798 PMC3583990

[15] Qian C., Lai C.J., Bao R., Wang D.G., Wang J., Xu G.X. (2012) Cancer network disruption by a single molecule inhibitor targeting both histone deacetylase activity and phosphatidylinositol 3-kinase signaling. Clin. Cancer Res. 18, 4104–4113 10.1158/1078-0432.CCR-12-0055 22693356

[16] Caiment F., Gaj S., Claessen S. and Kleinjans J. (2015) High-throughput data integration of RNA-miRNA-circRNA reveals novel insights into mechanisms of benzo[a]pyrene-induced carcinogenicity. Nucleic Acids Res. 43, 2525–2534 10.1093/nar/gkv115 25690898 PMC4357716

[17] Li H., Hao X., Wang H., Liu Z., He Y., Pu M. (2016) Circular RNA expression profile of pancreatic ductal adenocarcinoma revealed by microarray. Cell. Physiol. Biochem. 40, 1334–1344 10.1159/000453186 27997903

[18] Salzman J., Gawad C., Wang P.L., Lacayo N. and Brown P.O. (2012) Circular RNAs are the predominant transcript isoform from hundreds of human genes in diverse cell types. PLoS One 7, e30733 10.1371/journal.pone.0030733 22319583 PMC3270023

[19] Suto T., Yokobori T., Yajima R., Morita H., Fujii T., Yamaguchi S. (2015) MicroRNA-7 expression in colorectal cancer is associated with poor prognosis and regulates cetuximab sensitivity via EGFR regulation. Carcinogenesis 36, 338–345 10.1093/carcin/bgu242 25503932

[20] Rai K., Takigawa N., Ito S., Kashihara H., Ichihara E., Yasuda T. (2011) Liposomal delivery of MicroRNA-7-expressing plasmid overcomes epidermal growth factor receptor tyrosine kinase inhibitor-resistance in lung cancer cells. Mol. Cancer Ther. 10, 1720–1727 10.1158/1535-7163.MCT-11-0220 21712475

[21] Reddy S.D., Ohshiro K., Rayala S.K. and Kumar R. (2008) MicroRNA-7, a homeobox D10 target, inhibits p21-activated kinase 1 and regulates its functions. Cancer Res. 68, 8195–8200 10.1158/0008-5472.CAN-08-2103 18922890 PMC3636563

[22] Webster R.J., Giles K.M., Price K.J., Zhang P.M., Mattick J.S. and Leedman P.J. (2009) Regulation of epidermal growth factor receptor signaling in human cancer cells by microRNA-7. J. Biol. Chem. 284, 5731–5741 10.1074/jbc.M804280200 19073608

[23] Hansen T.B., Kjems J. and Damgaard C.K. (2013) Circular RNA and miR-7 in cancer. Cancer Res. 73, 5609–5612 10.1158/0008-5472.CAN-13-1568 24014594

[24] Memczak S., Jens M., Elefsinioti A., Torti F., Krueger J., Rybak A. (2013) Circular RNAs are a large class of animal RNAs with regulatory potency. Nature 495, 333–338 10.1038/nature11928 23446348

[25] Jakszyn P. and Gonzalez C.A. (2006) Nitrosamine and related food intake and gastric and oesophageal cancer risk: a systematic review of the epidemiological evidence. World J. Gastroenterol. 12, 4296–4303 10.3748/wjg.v12.i27.4296 16865769 PMC4087738

[26] Yang Q., Xu E., Dai J., Wu J., Zhang S., Peng B. (2014) miR-21 regulates N-methyl-N-nitro-N’-nitrosoguanidine-induced gastric tumorigenesis by targeting FASLG and BTG2. Toxicol. Lett. 228, 147–156 10.1016/j.toxlet.2014.05.005 24821435

[27] Liu Z., Li M., Fang X., Shen L., Yao W., Fang Z. (2019) Identification of surrogate prognostic biomarkers for allergic asthma in nasal epithelial brushing samples by WGCNA. J. Cell. Biochem. 120, 5137–5150 10.1002/jcb.27790 30304558

[28] Yi G., Liang M., Li M., Fang X., Liu J., Lai Y. (2018) A large lung gene expression study identifying IL1B as a novel player in airway inflammation in COPD airway epithelial cells. Inflamm. Res. 67, 539–551 10.1007/s00011-018-1145-8 29616282

[29] Lai Y., Liang M., Hu Z.Z., Lin H., Yi G., Li M. (2019) RNF135 is a positive regulator of IFN expression and involved in RIG-I signaling pathway by targeting RIG-I. Fish Shellfish Immunol. 86, 474–479 30508673 10.1016/j.fsi.2018.11.070

[30] Liu Z.Y., Jia K.T., Li C., Weng S.P., Guo C.J. and He J.G. (2013) A truncated Danio rerio PKZ isoform functionally interacts with eIF2alpha and inhibits protein synthesis. Gene 527, 292–300 10.1016/j.gene.2013.05.043 23742890

[31] Zhang Y., Zhang X.O., Chen T., Xiang J.F., Yin Q.F., Xing Y.H. (2013) Circular intronic long noncoding RNAs. Mol. Cell 51, 792–806 10.1016/j.molcel.2013.08.017 24035497

[32] Zhang X.O., Wang H.B., Zhang Y., Lu X., Chen L.L. and Yang L. (2014) Complementary sequence-mediated exon circularization. Cell 159, 134–147 10.1016/j.cell.2014.09.001 25242744

[33] Qian Y., Lu Y., Rui C., Cai M. and Jia R. (2016) Potential significance of circular RNA in human placental tissue for patients with preeclampsia. Cell. Physiol. Biochem. 39, 1380–1390 10.1159/000447842 27606420

[34] Cheng D.L., Xiang Y.Y., Ji L.J. and Lu X.J. (2015) Competing endogenous RNA interplay in cancer: mechanism, methodology, and perspectives. Tumour Biol. 36, 479–488 10.1007/s13277-015-3093-z 25604144

[35] Hentze M.W. and Preiss T. (2013) Circular RNAs: splicing’s enigma variations. EMBO J. 32, 923–925 10.1038/emboj.2013.53 23463100 PMC3616293

[36] Xiong S., Zheng Y., Jiang P., Liu R., Liu X. and Chu Y. (2011) MicroRNA-7 inhibits the growth of human non-small cell lung cancer A549 cells through targeting BCL-2. Int. J. Biol. Sci. 7, 805–814 10.7150/ijbs.7.805 21750649 PMC3133888

[37] Zhao X., Dou W., He L., Liang S., Tie J., Liu C. (2013) MicroRNA-7 functions as an anti-metastatic microRNA in gastric cancer by targeting insulin-like growth factor-1 receptor. Oncogene 32, 1363–1372 10.1038/onc.2012.156 22614005

[38] Saydam O., Senol O., Wurdinger T., Mizrak A., Ozdener G.B., Stemmer-Rachamimov A.O. (2011) miRNA-7 attenuation in Schwannoma tumors stimulates growth by upregulating three oncogenic signaling pathways. Cancer Res. 71, 852–861 10.1158/0008-5472.CAN-10-1219 21156648 PMC3072568

[39] Peeters M., Price T. and Van Laethem J.L. (2009) Anti-epidermal growth factor receptor monotherapy in the treatment of metastatic colorectal cancer: where are we today? Oncologist 14, 29–39 10.1634/theoncologist.2008-0167 19144681

[40] Hansen T.B., Wiklund E.D., Bramsen J.B., Villadsen S.B., Statham A.L., Clark S.J. (2011) miRNA-dependent gene silencing involving Ago2-mediated cleavage of a circular antisense RNA. EMBO J. 30, 4414–4422 10.1038/emboj.2011.359 21964070 PMC3230379

