# Supplementary material for: High-throughput sequencing reveals circular RNA hsa_circ_0000592 as a novel player in the carcinogenesis of gastric carcinoma
Source: Biosci Rep. 2019 Jun 28;39(6):BSR20181900. doi: 10.1042/BSR20181900 (PMC6597853; doi:10.1042/BSR20181900)
Supplement: Supplementary file 1 [file bsr20181900_Supp1.pdf]

Supplementary Table S1   RNA Primer sequences

Gene	Primer Sequence
hsa_circ_0000118	Forward-TTGCAAGGAAAGGACACTCC
	Reverse-CGTTCTTCCTCTTCCCTCCT
hsa_circ_0000284	Forward-TCGGCCAGTCATGTATCAAA
	Reverse-CCCTTAGTGGGAGGATGAGA
hsa_circ_0000567	Forward-CAAACCCTCCCCAGTGAATA
	Reverse-TCCTTTGGTGACACAGTTGC
hsa_circ_0000119	Forward-GGAGGCCTACTTGCAGCATA
	Reverse-GCTTCTTCCAAGGCCTTCTC
hsa_circ_0000847	Forward-TATTCCAGAAACGCCACCTC
	Reverse-TTCCATCCCAGCAGTCTCTT
hsa_circ_0000117	Forward-GGGCAAAGATGGATTGAAGA
	Reverse-AATAGGGACTGGTGGCAGTG
hsa_circ_0001829	Forward-GTGATGTTTGGCAGGGAGTT
	Reverse-TGGCTTGCTGGTTTACTGTG
hsa_circ_0002484	Forward- AAGTGTCCCAGCCTGTCAGT
	Reverse- AGCTTTCCAAGCATTTCCTG
hsa_circ_0000620	Forward- CTGAATGCCAATGTGTGGTC
	Reverse- CTATCAAGGCCCGATTTTTG
hsa_circ_0000592	Forward- CATGCAGTTGATGGGACAAC
	Reverse- TGAAGCTTCCGTTCTGGTCT
hsa_circ_0003221	Forward- GGGGCAATGCACTAGAAAAG
	Reverse- CCAGGTGGTTGGCTCACTAT
hsa_circ_0000268	Forward- TTGGCTCTTCCTCAATGCTT
	Reverse- TCTTTGGGCACGACACATAG
hsa_circ_0074990	Forward- ATCTTCGAGGGATTGCCTTT
	Reverse- GACACCTGAAGTCGCTGAGA
hsa_circ_0004594	Forward- GCGAGAAGCTCACCTTCAAC
	Reverse- CCAGAAGACCACCTTCTCCA
hsa_circ_0004873	Forward- ATGGTGACCTGGCTAACTGG
	Reverse- CTTCCCCATCTCCCTCTTTC
GAPDH	Forward-CAATGACCCCTTCATTGACC
	Reverse-GACAAGCTTCCCGTTCTCAG
U6	Forward –CTCGCTTCGGCAGCACA
	Reverse-AACGCTTCACGAATTTGCGT

Supplementary Table S2 siRNA sequences

siRNA	sequences
si-hsa_circ_0000592-1	sense 5'-AAUAAGACUUCUUGAAGAC-3',
	antisense 5'-GUCUUCAAGAAGUCUUAUU-3'
si-hsa_circ_0000592-2	sense 5'-AGAAGUCUUAUUGCCAGCA-3',
	antisense 5'-UGCUGGCAAUAAGACUUCU-3'
negative control siRNA	sense 5'-UUCUCCGAACGUGUCACGU-3',
	antisense 5'-ACGUGACACGUUCGGAGAA-3'

Supplementary Table S3

**circRNA h**sa**_circ_0000592**	**oligonucleotide probe sequence**
	TCTTATTGCCAGCAGTTTTGAAGATGACTCCCGTGTAGCCTCA
	CCGTTAGACCAGAACGGAAGCTTCAATGTTGTTATTAAAGAG
	GAACCTCTAGATGATTATGACTACGAACTTGGTGAGTGCCCAG
	AAGGGGTCACTGTGAAACAGGAAGAGACAGATGAAGAGACG
	GATGTATACTCAAACAGTGATGATGATCCTATACTAGAGAAAC
	AGCTAAAGAGGCACAATAAAGTTGACAACCCAGAAGCTGAC
	CATCTATCTTCTAAATGGCTTCCAAGCAGCCCATCAGGTGTTG
	CTAAAGCTAAAATGTTCAAATTAGACACTGGAAAGATGCCAGT
	AGTCTATCTGGAGCCCTGTGCTGTCACCAGAAGCACAGTTAA
	GATTTCTGAACTCCCCGATAACATGCTTTCCACATCTCGAAAG
	GATAAATCTTCTATGTTGGCAGAATTGGAATATTTGCCTACATA
	CATTGAAAATTCCAATGAGACTGCCTTCTGCTTAGGCAAGGAA
	TCAGAAAATGGTCTTAGAAAACATTCACCAGATCTCAGAGTG
	GTACAAAAATATCCCTTACTGAAAGAGCCTCAGTGGAAATATC
	CTGATATATCTGACAGCATTAGCACAGAAAGAATACTCGACGA
	TTCAAAGGATTCAGTTGGAGACTCACTTTCAGGAAAAGAGGA
	CTTGGGCAGAAAGAGAACAACTATGCTTAAGATTGCAACAGC
	CGCAAAGGTAGTGAATGCTAATCAGAATGCCTCTCCAAATGTC
	CCTGGAAAAAGAGGAAGGCCACGAAAATTGAAACTCTGTAA
	GGCAGGACGACCACCTAAGAACACAGGAAAGTCTTTAATTTC
	TACAAAGAATACACCTGTAAGCCCTGGGAGTACCTTTCCAGAT
	GTGAAGCCTGATCTGGAAGATGTGGATGGTGTTCTCTTTGTTT
	CCTTTGAATCAAAGGAAGCTCTAGACATTCATGCAGTTGATGG
	GACAACAGAAGAATCTTCTAGTCTCCAGGCATCAACCACAAA
	TGACTCAGGTTACAGAGCAAGAATTTCCCAGTTGGAAAAGGA
	ATTGATAGAAGATTTGAAGACTTTGCGGCACAAGCAGGTGAT
	ACATCCTGGTCTTCAAGAAG
